# Clinical trial and performance evaluation of the Wantai HBsAg (CMIA) diagnostic kit for screening blood donors in China

**DOI:** 10.1038/s41598-024-51910-1

**Published:** 2024-02-02

**Authors:** Jianfeng Chen, Fengtian Wang, Jiaxing Li, Qi Zuo, Dandan Wu, Chen Xiao

**Affiliations:** Shandong Blood Center, Jinan, 250014 Shandong China

**Keywords:** Immunology, Microbiology, Diseases, Medical research

## Abstract

In China, according to the ‘Technical Operating Procedures for Blood Stations (2019 Edition),’ blood stations are authorized to utilize Chemiluminescence Immunoassay (CLIA) to detect pathogen markers linked with transfusion-transmissible infections. However, currently, there is no approved CLIA reagent for the screening of blood-borne diseases in China, specifically for the detection of Hepatitis B surface antigen. The objective of this research project is to conduct a comprehensive evaluation of the performance of the Wantai Chemiluminescent Microparticle Hepatitis B surface antigen reagent. This study evaluates the performance of the Wantai Chemiluminescent Microparticle Immunoassay (CMIA) on the Wan200 + analyzer in screening for Hepatitis B Surface Antigen (HBsAg) in blood samples. The clinical trial component of this evaluation is included as part of the documentation submitted to the National Medical Products Administration (NMPA) of China for the approval of blood screening reagents. The evaluation plan of this study encompasses two main components: clinical trials and performance assessment. We adopted a controlled trial design, utilizing the WanTai Chemiluminescent Microparticle Immunoassay (CMIA) on the Wan200 + analyzer and the Enzyme-Linked Immunosorbent Assay (ELISA) to screen for Hepatitis B Surface Antigen (HBsAg) in routine blood donor samples and reference serum panel samples. To ensure the accuracy of the screening, we additionally employed Abbott's ELISA reagents and HBV DNA for validation. The assessment primarily focused on key performance indicators such as sensitivity, specificity, and analytical sensitivity. Moreover, this clinical trial data has been included as part of the submission to China's National Medical Products Administration (NMPA). In the clinical trials of this study, a total of 10,470 blood donor samples underwent simultaneous testing using both CMIA and ELISA methods. Across two clinical trials, there was remarkable concordance between CMIA and the two ELISA reagents, with Kappa values exceeding 0.82. Among the 269 samples that were double-reactive in the enzyme immunoassay (ELISA) tests, CMIA exhibited a 100% reactivity detection rate. However, CMIA produced 14 and 6 false-positive results in the respective clinical trials, resulting in specificities of 99.73% and 99.89%. In contrast, the specificities for Wantai ELISA and Xin Chuang ELISA were both greater than 99.94%.When testing samples in the gray zone serum plates, CMIA's detection limit significantly exceeded that of the two ELISA assays. CMIA had a detection cutoff of 0.05 IU/mL, while the two ELISA reagents had cutoffs of 0.1 IU/mL and 0.09 IU/mL, respectively. CMIA's detection limits for the adr and adw subtypes were 0.05 IU/mL, and for the ay subtype, it was 0.1 U/mL. The detection limit for 10 HBV mutant samples was 0.5 U/mL. In 165 cases where ELISA tested negative but HBV DNA tested positive, CMIA detected 5 HBsAg-positive samples. This study evaluated the performance of the Wantai CMIA in screening for HBsAg among blood donors. The results demonstrate outstanding performance of CMIA in both clinical trials and performance assessments, detecting all true positive samples with a sensitivity of 100%. It exhibits excellent concordance with the two ELISA assays. Of particular note is its superiority in early detection of HBsAg in the screening of early-stage hepatitis B infections, reducing the window period compared to ELISA. CMIA achieves a specificity exceeding 99.73% for negative blood donors, aligning with the European Union's standards for blood screening assay specificity. In summary, Wantai's CMIA displays high sensitivity and specificity in blood donor screening, making it suitable for screening blood donors in China.

## Introduction

The Hepatitis B virus (HBV) can result in acute or chronic viral hepatitis type B. HBV is primarily transmitted through the mother-to-child route, especially during birth and delivery. Additionally, contact with contaminated blood or other bodily fluids during sexual intercourse with an infected individual, as well as transmission through unsafe injections or exposure to contaminated sharp objects, are also common modes of viral transmission. According to estimates by the World Health Organization, approximately 296 million individuals worldwide were living with chronic Hepatitis B infection in 2019, with an annual incidence of 1.5 million new infections. In the same year, Hepatitis B was responsible for around 820,000 deaths, with most fatalities attributed to cirrhosis and primary liver cancer. Effective prevention of Hepatitis B infection can be achieved through the administration of safe, accessible, and effective vaccines^1^. According to estimates, approximately 90 million people in China were living with chronic hepatitis B (HBV) in 2015. Among them, 28 million individuals required treatment, and 7 million individuals urgently needed treatment due to advanced liver disease and the high risk of developing cancer^2^. To prevent the transmission of HBV through blood transfusion, China currently employs the enzyme-linked immunosorbent assay (ELISA) method for HBsAg detection. The “Technical Operating Procedures for Blood Stations (2019 Edition)” indicates that chemiluminescence immunoassay (CLIA) can be used for blood screening^3^. Currently, no manufacturer's CLIA reagent has obtained approval from the China National Medical Products Administration (NMPA) for blood donor screening. In accordance with the requirements of the “Regulations for the Registration and Administration of In-vitro Diagnostic Reagents” and the “Technical Operating Procedures for Blood Stations (2019 Edition),” this study evaluated the application of the Wantai HBsAg CMIA reagent kit for blood screening and compared it with the ELISA reagent kit.

## Materials and methods

### Specimens

#### Clinical trial samples

In the clinical trial group, a total of 10,470 samples were included, consisting of samples from routine blood donors (5,151 samples tested from August 19 to August 31, 2021, and 5,319 samples tested from May 8 to May 20, 2023). This group also included previously retained samples that tested reactive with two ELISA assays. In the clinical trial section of this study, samples that tested positive with both ELISA assays simultaneously were considered true positives. The specific sample types are detailed in Table 1.

**Table 1:** Composition of Clinical Trial Samples.

Sample type and quantity	Number
HBsAg Pos samples	56
HIV Ag/Ab Pos samples	25
HCV-Ab Pos samples	30
TP-Ab Pos samples	26
Routine blood donor samples	10,333
Total	10,470

In this table, "HBsAg positive Samples" signifies the quantity of samples that produced positive results in both preceding ELISA dual-reagent tests, with the exception of the count of HBsAg positives identified during Routine Blood Donor Samples testing.

#### Reference Serum Samples

A total of 554 reference serum samples were included, comprising samples that reacted positively with both ELISA assays for HBsAg, samples with different subtypes of HBsAg, mutation samples, equivocal samples, and HBV DNA-positive samples. Detailed information about the samples can be found in Table 2.

**Table 2:** Reference Serum Samples.

Sample types	Number
HBsAg Pos samples	269
Subtype reference serum samples	20
Mutation reference serum samples	40
Equivocal Neg and Pos Samples	60
HBsAg Neg/HBV DNA Pos Samples	165
Total	554

### Experimental sample requirements

#### ELISASample collection and handling

Clinical samples used in this study were residual plasma samples obtained from routine laboratory testing. The overall sample quality requirements were as follows: If a sample appeared turbid or contained sediment, it underwent centrifugation or filtration to obtain the supernatant suitable for testing. Additionally, samples needed to be free of microbial contamination and should not contain suspended fibrinogen or aggregates that would impede testing.

#### ELISASample storage conditions

Samples intended for testing within a period of up to 7 days were stored at temperatures between 2 °C and 8 °C. For long-term storage, samples were maintained at temperatures below −20°C.

#### ELISAReference serum samples

Reference serum samples were stored at temperatures of − 30°C from the date of collection, and they were not thawed during the experimental period.

### Inclusion and exclusion criteria

#### Inclusion criteria

Samples must meet the requirements for experimental sample grouping; clinical trial data sheets must contain complete and clearly traceable information; experimental samples must not contain suspended fibrinogen or aggregates and should not exhibit severe hemolysis; estimated residual sample volume should be approximately 900μL; samples must meet the requirements for sample storage conditions and freeze–thaw cycles. Samples that meet all five of these requirements are eligible for inclusion in this trial, while those that do not meet the criteria should be excluded.

#### Exclusion criteria

Samples that were not tested strictly according to the clinical protocol and instructions for each test reagent; samples with insufficient volume to complete the test; other samples that do not meet the requirements of the clinical trial. If any of the three criteria mentioned above are met, the sample is considered invalid and should be excluded.

### Interpretation of test results

Enzyme immunoassay results are interpreted based on the manufacturer's reagent instructions, calculating the S/CO value. A result with S/CO ≥ 1 is considered positive, while a result with S/CO < 1 is considered negative. CMIA test results are automatically determined using the Wan200 + analyzer, which compares the electrochemiluminescence signal from the sample with the calibrated cutoff value. Samples with a cutoff index (COI) less than 1.0 are non-reactive and considered negative without further testing. Samples with a COI of 1.0 or higher are considered reactive. For clinical trials, positive samples from routine blood donors are retested twice. In any repeat test, a result with S/CO ≥ 1.0 is considered reactive, otherwise, it is considered negative. Serum plates are tested only once, and the initial result is considered final.

#### Confirmation testing

In case of discrepancies, reference is made to results from a third-party reagent (Abbott ELISA) and analysis of NAT results. Results are interpreted as follows: if both the third-party reagent and NAT results are non-reactive, the result is considered negative; if the third-party reagent is non-reactive but NAT is reactive, the result is considered positive; if the third-party reagent is reactive but NAT is non-reactive, the result is considered indeterminate.

### Reagents and instruments

The chemiluminescence reagent used in this study is the Wantai Diagnostic Kit for Hepatitis B Virus Surface Antigen (CMIA). The comparative enzyme immunoassay reagents include the Hepatitis B Virus Surface Antigen Diagnostic Kit (ELISA) by Beijing Wantai (referred to as WT ELISA), and the third-party ELISA reagents by Livzon or Xinchuang. For the ELISA method, sample addition was carried out using the Hamilton Microlab STAR BG and STAR Venus 8CH instruments. Incubation, plate washing, and absorbance measurement were conducted using the FAME24/20 and FAME24/30 instruments. The Wan200 + CMIA instrument was used for the chemiluminescence immunoassay (CLIA) method.

#### Principles of the CMIA method

The HBsAg in human serum and plasma is detected using the principle of double-antibody sandwich method (chemiluminescent magnetic microparticles). Firstly, the sample, antibody-coated magnetic microparticles, and reaction diluent are mixed. The antigen in the sample binds to the antibody-coated magnetic microparticles. After magnetic separation and washing to remove unbound substances, the fluorescein-labeled antibody is added to form a "antibody-coated magnetic microparticles —HBsAg— fluorescein-labeled antibody" complex. After magnetic separation and washing, the pre-triggering solution and triggering solution are added. The complex emits a light signal whose intensity can be detected by a luminometer and represented as relative light units (RLU). The intensity of the light signal is directly proportional to the amount of HBsAg in the sample. The instrument calculates the S/CO (COI) value of HBsAg in the sample based on the calibration result.

### Gray zone verification serum plate studies

The gray zone verification serum plate comprises 60 samples, covering a concentration range from 0 IU/mL (negative) to 1 IU/mL. It's worth noting that 1 IU/mL is equivalent to 20 COI. Two of the samples have values of 0.05 IU/mL, which is equivalent to 1 COI and a cutoff of 1. Results greater than or equal to 1 are defined as positive, while those below 1 are considered negative. The primary objective of the gray zone verification serum plate is to validate the rationality of the CUTOFF value for the HBsAg assay and ensure its compliance with practical requirements. This helps enhance result accuracy, reduce the risk of misclassification, and ensure a clear differentiation between true positive and true negative results.

### Statistical analysis

#### Clinical trial

Statistical analysis was performed on the results of CMIA reagent and ELISA reagent testing in routine blood donors. Metrics such as positive agreement rate, negative agreement rate, overall agreement rate, Kappa value, were calculated. Sensitivity and specificity were analyzed using SPSS 22.0 statistical software, employing the chi-squared test. A significance level of *P* < 0.01 indicated statistically significant differences.

#### Sensitivity calculation

We define true-positive samples as those that tested reactive in both ELISA assays and samples with only one ELISA assay testing positive, but with a positive result in the HBV DNA test. Then, we calculate the sensitivity of a particular assay by dividing the number of true-positive samples detected by that assay by the total number of true-positive samples.

#### Specificity calculation

Specificity refers to all samples other than true-positive ones, including those correctly identified as negative in both assays or samples that tested positive in one assay but had a negative HBV DNA result. We calculate the specificity of a particular assay by dividing the number of true-negative samples detected by that assay among all negative samples.

### Detection process diagram

**Figure 1:**
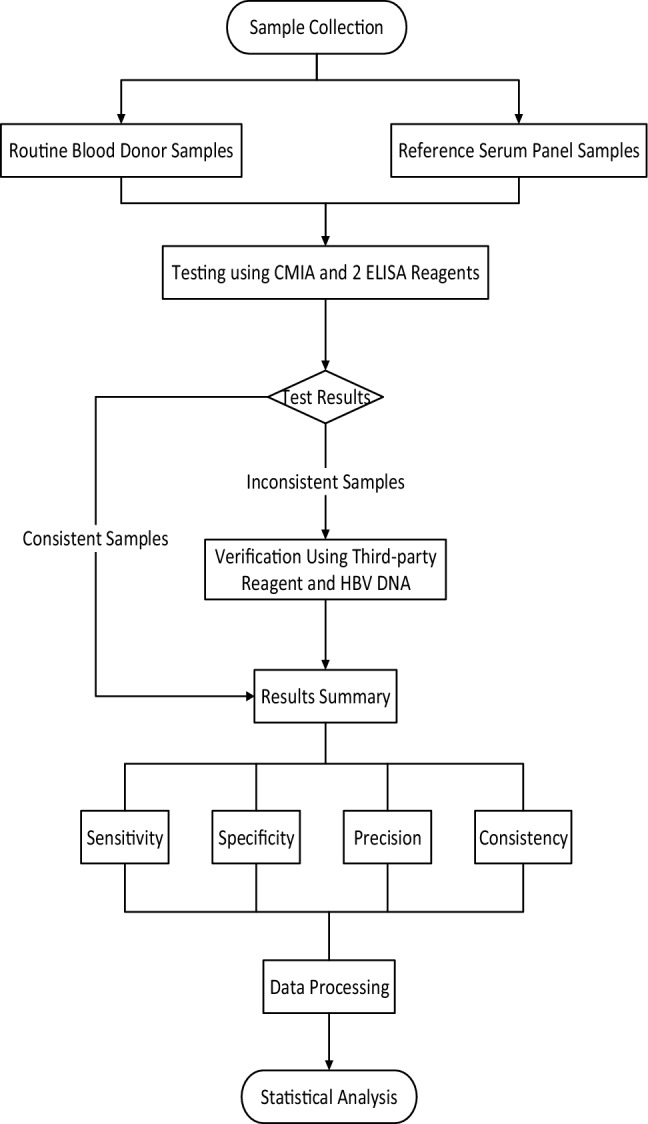
HBsAg Detection Process.

The detection process diagram for the clinical trial can be found in Fig. 1.

### Ethical approval and informed consent

In this study, all participating blood donors had signed the “Blood Donor Informed Consent Form” and filled out the “Health Status Inquiry Form” before donating blood. The consent form clearly outlined the purpose of the donation, related procedures, potential risks, and confidentiality measures. Donors voluntarily gave their consent after fully understanding these aspects. Regarding the ethics review, all methods described in this study followed relevant guidelines and regulations. The experimental protocol was approved by the “Ethics Committee of the Shandong Blood Center (Medical and Health Technology Development Program of Shandong Province).” Additionally, the chemiluminescent clinical trial mentioned in our manuscript adhered to the ethical principles of the Declaration of Helsinki and the relevant regulations and laws of the China National Medical Products Administration. This trial was conducted in vitro, using surplus plasma and serum samples from routine testing at the clinical trial institution, thus eliminating the need for additional sample collection. These samples would not cause any foreseeable harm to the participants, and the results would not affect their medical decisions or standard diagnosis and treatment. All submitted data, including participant information, were anonymized using sample codes to ensure the highest level of privacy and protection of personal information.

## Results

### Clinical trial results

In this clinical trial for HBsAg, a total of 10,470 samples were tested. The reagent batch numbers for the Wantai CMIA used in 2021 were 20,210,301 for the reagent, 30,210,704 for the pre-trigger solution, and 30,210,402 for the triggering solution. In 2023, the reagent batch numbers were 30,230,102 for the reagent, 30,221,214 for the pre-trigger solution, and 30,221,213 for the triggering solution.

All three reagents exhibited a 100% detection rate for the 59 previously retained ELISA double-reactive positive samples. In the first clinical trial, there was a statistically significant difference in specificity between WT CMIA and Wantai ELISA reagents, but in the second clinical trial, there was no significant difference between them. There was also no significant difference in specificity between WT CMIA and the Xinchuang ELISA reagent. Tables 3, 4 and 5 show the clinical trial results for the year 2021, while Tables 6, 7 and 8 display the clinical trial results for the year 2023.

**Table 3:** A 2021 CMIA vs. WT ELISA HBsAg Test Results.

		WT ELISA		Total
		Pos	Neg	
CMIA	Pos	37	14	51
	Neg	2	5098	5100
Total		39	5112	5151

**Table 4:** A 2021 CMIA vs. XC ELISA HBsAg Test Results.

		XC ELISA		Total
		Pos	Neg	
CMIA	Pos	39	12	51
	Neg	1	5099	5100
Total		40	5111	5151

CMIA and XC ELISA both showed 2 false positive samples in common.

**Table 5:** Clinical Trial Results for HBsAg in 2021 (n = 5151).

Donor samples	WT CMIA	WT ELISA	XC ELISA
Total samples	5151		
Pos with 3 assays	37	37	37
False Pos	14	2	3
Neg	5100	5112	5111
Sensitivity	100%	100%	100%
Specificity	99.73%	99.96%	99.94%
*P*-value		< 0.01	> 0.01
Pos Agreement Rate		94.87%*	97.50%*
Neg Agreement Rate		99.73%*	99.77%*
Overall agreement rate		99.69%*	99.75%*
Kappa value		0.82*	0.856*

1. *P-value, Positive Agreement Rate, Negative Agreement Rate, Overall Agreement Rate, Kappa Value are all comparisons between ELISA and CMIA.

2. The difference in specificity between CMIA and Wantai ELISA is statistically significant, *χ*^2^ = 7.574, *P* = 0.006 (continuity corrected); no significant difference was observed between CMIA and Xinchuang ELISA, *χ*^2^ = 5.892, *P* = 0.015 (continuity corrected).

3. Wantai CMIA and Xinchuang ELISA both identified 2 false-positive samples in their detection results.

**Table 6:** A 2023 CMIA vs. WT ELISA HBsAg Test Results.

		WT ELISA		Total
		Pos	Neg	
CMIA	Pos	22	6	28
	Neg	1	5290	5291
Total		23	5296	5319

**Table 7:** A 2023 CMIA vs. XC ELISA HBsAg Test Results.

		XC ELISA		Total
		Pos	Neg	
CMIA	Pos	22	6	28
	Neg	0	5291	5291
Total		22	5297	5319

**Table 8:** A 2023 Clinical Trial Results for HBsAg (n = 5319).

Donor samples	WT CMIA	WT ELISA	LZ ELISA
Total samples	5319		
Pos with 3 assays	22	22	22
False Pos	6	1	0
Neg	5291	5296	5297
Sensitivity	100%	100%	100%
Specificity	99.89%	99.98%	100%
*P*-value		> 0.01*	> 0.01*
Pos Agreement Rate		95.65%*	100%*
Neg Agreement Rate		99.89%*	99.89%*
Overall Agreement Rate		99.87%*	99.89%*
Kappa Value		0.86*	0.88*

1. *P values, Positive agreement rate, Negative agreement rate, Overall agreement rate, and Kappa values are all comparisons between ELISA and CMIA.

The difference in specificity between CMIA and WTELISA is not statistically significant, χ2 = 2.287, P = 0.130 (continuity-corrected), and there is also no significant difference compared to XinChuang ELISA, P = 0.031 (Fisher's exact probability method).

### Detection results for HBsAg-Positive and HBsAg-Negative/HBV DNA-Positive samples

“HBsAg positive” refers to both ELISA assays yielding positive results, while “HBsAg-negative/HBV DNA-positive samples” indicates that both ELISA assays yielded negative results. CMIA detection results are presented in Table 9.

**Table 9:** HBsAg Positive and HBsAg-Negative/HBV DNA Positive Sample Detection Results.

	Sample count	CMIA positive detection
CMIA Pos CountHBsAg positive	269	269
HBsAg-Negative/HBV DNA +	165	5

### Detection results for interference samples

When testing the 81 interference samples, both CMIA and ELISA reagents identified them as Negative, achieving a 100.00% Negative agreement rate. There were no instances of cross-reactivity, indicating that CMIA reagents have a high interference resistance. See Table 10 for details.

**Table 10:** Detection Results of Assessment Reagents and Comparative Reagents for Interference Samples.

Sample group	Number	CMIA Neg	WT ELISA Neg	Specificity (%)
HIV Antibody Pos Samples	25	25	25	100.00%
HCV Antibody Pos Samples	30	30	30	100.00%
TP Antibody Pos Samples	26	26	26	100.00%
Total	81	81	81	100.00%

The data in this table is already included in the clinical trial Table 4.

### Gray zone verification serum plate test results

There were a total of 60 Gy zone verification serum plate samples, with HBV content ranging from 0 to 1 IU/ml, and COI values ranging from 0 to 20 COI, where 1 IU/ml equals 20 COI. These two graphs display data for different parameters in the serum plate samples, including HBV content in IU/ml, standard COI values in serum plate samples, CMIA COI values, WT ELISA S/CO, and XC ELISA S/CO. Among these parameters, except for two samples with 0.00 IU/ml, all other samples had CMIA COI values higher than both ELISA S/CO values.

**Figure 2:**
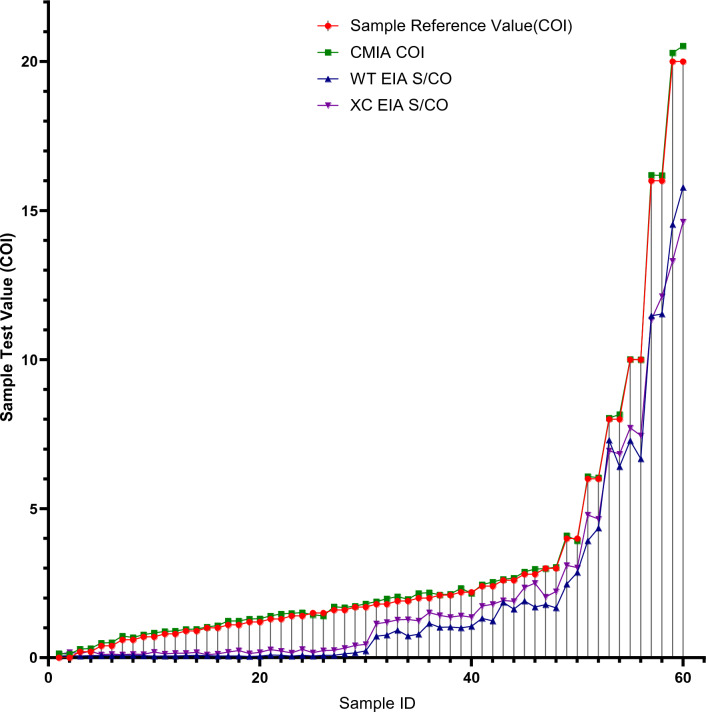
Gray Zone Sample Detection Results.

**Figure 3:**
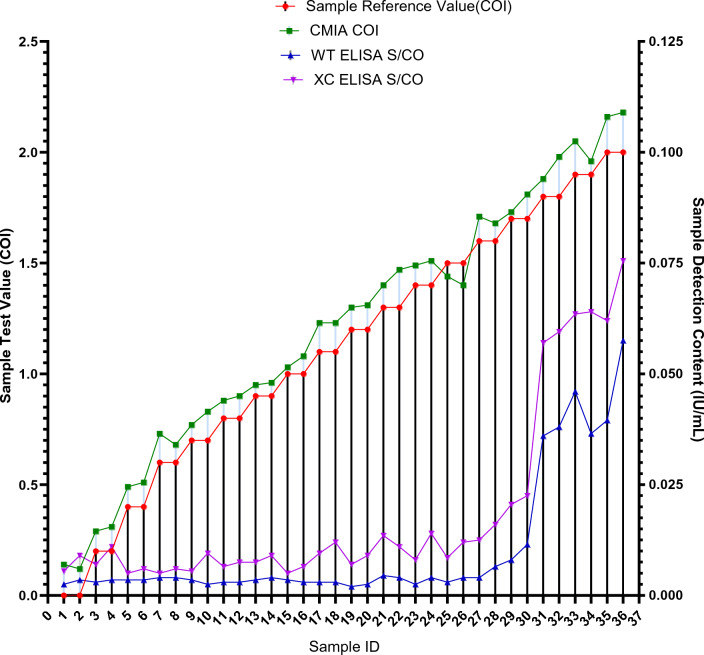
Comparison Chart of Gray Zone Low-Value Sample Test Results.

From the test data, it can be observed that the first 14 samples were Negative for all three assays. However, starting from the 15th sample, CMIA detected a Positive result. At this point, the HBV content in the serum plate reference sample was 0.5 IU/ml, with a detection value of 1.00 COI. This indicates that CMIA has a higher threshold for S/CO values compared to ELISA and is more capable of detecting samples with low HBV loads. The comparison of the detection results for all three assays against the standard values in the serum plate is shown in Fig. 2, while the results for the 36 low-value samples are presented in Fig. 3.

### Subtype detection results

CMIA tested different HBsAg subtypes at five different dilution ratios, demonstrating a 100% detection rate for the three main Chinese HBV subtypes with a concentration of 0.05 IU/mL.The results of the tests can be found in Table 11.

**Table 11:** CMIA detection results for 3 different HBV subtypes at various dilution concentrations.

Number	Subtype	Serum Disk (IU/mL)	Serum Disk (COI)	Interpretation
1	adr	0.05	1.08	( +)
2	adr	0.1	1.59	( +)
3	adr	0.2	3.19	( +)
4	adr	0.5	7.91	( +)
5	adr	1	15.97	( +)
6	adw	0.05	1.14	( +)
7	adw	0.1	1.74	( +)
8	adw	0.2	3.38	( +)
9	adw	0.5	8.72	( +)
10	adw	1	16.86	( +)
11	ay	0.05	1.81	( +)
12	ay	0.1	3.08	( +)
13	ay	0.2	7.33	( +)
14	ay	0.5	15.55	( +)
15	ay	1	29.94	( +)
16	NC	Neg	0.1	( −)
17	NC	Neg	0.12	( −)
18	NC	Neg	0.1	( −)
19	NC	Neg	0.1	( −)
20	NC	Neg	0.17	( −)

### Mutation detection results

CMIA tested different HBsAg mutation samples at three different dilution ratios, demonstrating a 100% detection rate for all ten different mutation points with a concentration of 0.5 U/mL.The results are available in Table 12.

**Table 12:** Mutation Detection Results—CMIA Results.

Number	HBsAg mutation sites	Serum disk U/mL	Serum Disk (COI)	Interpretation
1	G145A	0.5	3.97	( +)
2	G145A	1	7	( +)
3	G145A	5	14.71	( +)
4	M204R	0.5	2.86	( +)
5	M204R	1	6.35	( +)
6	M204R	5	12.51	( +)
7	K141E	0.5	5.84	( +)
8	K141E	1	11.49	( +)
9	K141E	5	23.13	( +)
10	C139R	0.5	6.44	( +)
11	C139R	1	11.61	( +)
12	C139R	5	24.9	( +)
13	D144R	0.5	2.93	( +)
14	D144R	1	5.86	( +)
15	D144R	5	11.44	( +)
16	P120T	0.5	4.56	( +)
17	P120T	1	7.8	( +)
18	P120T	5	17.13	( +)
19	Q129R	0.5	4.54	( +)
20	Q129R	1	8.8	( +)
21	Q129R	5	17.99	( +)
22	I126S	0.5	4.88	( +)
23	I126S	1	9.87	( +)
24	I126S	5	18.86	( +)
25	M204I	0.5	4.87	( +)
26	M204I	1	9.36	( +)
27	M204I	5	19.13	( +)
28	T140I	0.5	4.34	( +)
29	T140I	1	8.47	( +)
30	T140I	5	17.5	( +)
31	NC1	Neg	0.11	( −)
32	NC2	Neg	0.16	( −)
33	NC3	Neg	0.11	( −)
34	NC4	Neg	0.1	( −)
35	NC5	Neg	0.17	( −)
36	NC6	Neg	0.11	( −)
37	NC7	Neg	0.09	( −)
38	NC8	Neg	0.09	( −)
39	NC9	Neg	0.1	( −)
40	NC10	Neg	0.1	( −)

## Discussion

CMIA has replaced ELISA for HBsAg blood screening in many countries, such as the United States^4^, India^5^ and Pakistan]^6^. However, in China, no chemical luminescence reagent company has yet qualified for blood screening reagents. This study aimed to evaluate the performance of the Wantai CMIA in screening for HBsAg in blood. The research results indicate that CMIA detected 56 positive blood donors in clinical trials and also identified 296 positive samples, 15 subtypes, and 30 mutated HBsAg samples when compared to reference serum samples, all with a sensitivity of 100%. Furthermore, among 10,411 negative blood donors, CMIA showed a specificity of 99.81%, meeting the European Union standards for blood screening reagents' specificity at 99.50%^7^. These results demonstrate that CMIA reagents possess high sensitivity and specificity in blood screening for HBsAg, which has also been confirmed in other studies, such as Roche Elecsys^8,9^.

However, despite CMIA reagents exhibiting high specificity in clinical trials, 20 false-positive samples were observed, whereas ELISA detected only 3 false-positive samples, making CMIA's false-positive rate 6.7 times that of ELISA. This phenomenon may be attributed to various factors, including reagent preparation processes and COI (Cut-off Index) settings. To address this, we conducted a comprehensive study using grey area sample serum plates and samples that were HBV DNA positive but ELISA negative for validation. Our research revealed that CMIA uses different criteria for determining the cutoff value compared to ELISA. According to the standard criteria, the Limit of Quantification (LoQ) of the assay was determined to be 0.050 IU/mL, consistent with the Roche Elecsys HBsAg II Quant Assay^10^ and The Architect CLMIA by Abbott^11^. While CMIA interprets results with a COI value ≥ 0.05 as positive, ELISA requires an S/CO value ≥ 0.09 for a positive result. Therefore, the inconsistency in cutoff value standards between CMIA and ELISA is a significant factor contributing to the high false-positive rate of CMIA. Assuming CMIA adopts the cutoff value criteria of ELISA, i.e., positive for COI greater than 1.8, then among these 18 false positives, 11 would be classified as negative, leaving only 9 as positive, reducing the false-positive rate by 55%. This value is lower than the specificity of Elecsys® HBsAg II (100%, 3336/3336)^12^, but higher than that of the Automated Fluorescent Immunoassay System (AFIAS; Boditech Med Inc., Chuncheon, Korea) at 99.30% (1092/1100)^13^.

According to American literature, CLIA reduces the window period (WP) between HBsAg tests with permits by 2–7 days^14^. In this study, CMIA detected 5 HBsAg-positive cases among 165 samples that were HBV DNA positive but ELISA negative, confirming that CMIA can detect HBsAg earlier, thus shortening the "window period" in traditional methods. This is particularly important for samples with low viral loads, where CMIA's high sensitivity can prevent cases of missed detection.

A study by the Shenzhen Blood Center in China indicated that some reagents may miss low HBV load and mutated HBV strains^15^. Therefore, it is advisable to select reagents that are less affected by HBsAg mutations^16–21^. CMIA stands out for its high sensitivity for HBsAg and noteworthy is its higher sensitivity and lower detection limit compared to other testing methods.

In conclusion, the Wantai chemiluminescence microparticle immunoassay (CMIA) demonstrates high sensitivity and specificity in blood screening for HBsAg in blood donors, making it suitable for routine blood screening procedures.

## Data Availability

We acknowledge the journal's requirement for data availability. However, the data in our manuscript is currently undergoing submission to the China National Medical Products Administration (NMPA) as part of the approval process for blood screening reagents. Consequently, it is not advisable to make this data publicly accessible at this moment. Nevertheless, for the benefit of the reviewing experts and to facilitate their understanding of CMIA's testing performance, we have included the clinical data related to CMIA in the “Related Files” section. Rest assured, we will make the full dataset available once the regulatory approval process is completed during the review period.

## Abbreviations

ELISA: Enzyme-linked immunosorbent assay
HBV: Hepatitis B virus
PCR: Polymerase chain reaction
DNA: Deoxyribonucleic acid
NAT: Nucleic acid testing
S/CO: Signal to cut-off
WanTai/WT: WanTai BioPharm
LivZon/LZ: Zhuhai Livzon Diagnostics Inc
InTec/XC: InTec PRODUCTS,INC
Positive: Pos
Negative: Neg
